# Supplementary material for: The Disparity in Mental Health Between Two Generations of Internal Migrants (IMs) in China: Evidence from A Nationwide Cross-Sectional Study
Source: Int J Environ Res Public Health. 2019 Jul 22;16(14):2608. doi: 10.3390/ijerph16142608 (PMC6678572; doi:10.3390/ijerph16142608)
Supplement: Supplementary file 1 [file ijerph-16-02608-s001.pdf]

Table S1. Specification of social integration variables.

Variables	Definition
Integration will	Integration will consists of 13 questions, such as "I would like to live together with locals in a community", "I would like to be a colleague with locals" and "I would like to be neighbor with locals". Respondents were asked about the level of agreement with these statements based on four point scales (1 as disagree completely and 4 as agree completely) and the higher score means better integration will.
Views about social norms adopted	Those views include 8 questions about social norms: 1) The customs at hometown (such as the customs of marriage, funerals) is more important to the subject; 2) Working in the current place is more important to me than living at hometown; 3) The subject's child should learn to speak hometown dialect; 4) Maintaining the hometown's lifestyle, such as eating habits, is important; 5) There is a big difference on health habits between the subject and local residents; 6) There is a big difference on clothing between the subject and local residents; 7) There is a big difference on education or retirement style between the subject and local residents; 8) The subject's opinions of some social problems are very different from the local residents'. Respondents were asked to report their agreements with these views based on five point scale (strongly agree, agree, neither agree or not, disagree, strongly disagree).
Income, occupation position compared with the people of the whole society	Subjective social status and level of respect compared to the other people which was measured by marking a "social ladder" (1 as the bottom status to 10 as the top status).
Degree of respect compared with relatives, friends and colleagues of the current residence	
Willing to live in current residence for the next 5 years	Whether to live in current residence in the next 5 years.
Type of neighbours	Whether the neighbours of the subjects were registered residents, who had "*Hukou*", or migrants.
Thinking oneself native or not	Whether the subjects think themselves as native residents, who had "*Hukou*", or migrants.

Table S2. Differences in mental health between new and old generation migrants of different subgroups, 2014.

Characteristics	Psychological Distress			Perceived Stress		
	New Generation (n=9838)	Old Generation (n=6161)	Total[a] (n=15999)	New Generation (n=9838)	Old Generation (n=6161)	Total[a] (n=15999)
County-level						
GDP per capita *r*	<0.01	0.05	0.02*	-0.02	0.06	0.01
Individual-level						
Age *r*	-0.03	-0.04	-0.05***	-0.05	-0.03	-0.06***
Weekly working hours *r*	-0.03	-0.01	-0.02**	0.01	-0.02	-0.01
Annual income/Regional GDP per capita 2013 *r*	-0.02	-0.04	-0.03**	-0.04	-0.04	-0.05***
Sex mean(SD)						
Male	3.5(3.1)	3.2(3.0)	3.5(3.0)***	5.4(2.6)	5.1(2.7)	5.3(2.6)***
Female	3.5(3.0)	3.3(3.0)	3.4(3.1)*	5.4(2.6)	5.3(2.7)	5.4(2.6)
Marital status mean(SD)						
Married	3.4(3.1)	3.2(3.0)	3.3(3.1)	5.2(2.6)	5.1(2.7)	5.2(2.6)**
Single	3.8(3.1)	3.8(3.1)	3.8(3.1)	5.6(2.6)	5.7(2.8)	5.6(2.6)
Education level mean(SD)						
Primary school and less	3.6(3.1)	3.2(3.0)	3.3(3.0)	5.7(2.5)	5.2(2.7)	5.3(2.7)**
Secondary school	3.4(3.0)	3.2(3.0)	3.3(3.0)**	5.4(2.6)	5.1(2.7)	5.3(2.6)***
High school	3.6(3.1)	3.5(3.3)	3.6(3.2)	5.5(2.6)	5.2(2.7)	5.4(2.6)**
College and above	3.7(3.2)	3.4(3.0)	3.7(3.2)	5.2(2.6)	5.2(2.7)	5.2(2.6)
Self-rated physical health mean(SD)						
Good	3.4(3.0)	3.1(2.9)	3.3(3.0)***	5.3(2.6)	5.0(2.6)	5.2(2.6)***

Fair	5.2(3.6)	4.3(3.4)	4.7(3.5)***	6.8(2.5)	6.1(2.6)	6.4(2.6)***
Poor	6.0(4.5)	5.9(4.3)	5.9(4.3)	7.5(2.3)	7.2(3.2)	7.3(2.9)
Duration of migration (months) *r*	-0.04	-0.05	-0.05***	-0.04	-0.09	-0.07***
Migration path mean(SD)						
Rural-Urban	3.6(3.1)	3.2(3.0)	3.5(3.1)***	5.5(2.6)	5.1(2.7)	5.3(2.6)***
Urban-Urban	3.8(3.4)	3.4(3.3)	3.6(3.4)*	5.4(2.7)	5.1(2.7)	5.3(2.7)*
Urban-Rural	3.3(2.8)	3.3(2.9)	3.3(2.9)	5.2(2.6)	5.2(2.7)	5.2(2.69)
Rural-Rural	3.5(3.1)	2.9(3.0)	3.2(3.1)	5.2(2.8)	5.0(3.0)	5.1(2.9)
Migrating with families mean(SD)						
No	3.8(3.1)	4.0(3.3)	3.8(3.1)	5.6(2.6)	6.0(2.9)	5.7(2.6)
Yes	3.4(3.1)	3.3(3.0)	3.3(3.0)*	5.3(2.6)	5.1(2.7)	5.2(2.6)
Integration will *r*	-0.12	-0.13	-0.13***	-0.13	-0.17	-0.14***
Views about social norms adopted *r*	-0.11	-0.13	-0.12***	-0.11	-0.13	-0.11***
Income, occupation position compared with the people of the whole society *r*	-0.16	-0.15	-0.15***	-0.18	-0.17	-0.18***
Degree of respect compared with relatives, friends and colleagues of the current residence *r*	-0.16	-0.15	-0.16***	-0.21	-0.23	-0.22***
Willing to live in current residence in the next 5 years mean(SD)						
Yes	3.4(3.1)	3.1(2.9)	3.3(3.0)***	5.1(2.6)	4.9(2.7)	5.0(2.7)***
No	3.8(3.2)	3.7(2.9)	3.8(3.1)	5.7(2.5)	5.6(2.5)	5.7(2.5)
Not sure	3.6(3.0)	3.6(3.3)	3.6(3.1)	5.7(2.5)	5.7(2.6)	5.7(2.6)
Type of neighbours mean(SD)						
Outsiders	3.6(3.1)	3.5(3.2)	3.6(3.1)*	5.5(2.6)	5.4(2.7)	5.5(2.6)*
The locals	3.3(3.1)	3.0(2.9)	3.2(3.0)**	5.0(2.6)	4.8(2.6)	4.9(2.6)*
Mixed	3.5(3.0)	3.2(3.0)	3.4(3.0)***	5.5(2.6)	5.1(2.7)	5.3(2.6)***
Think oneself native or not mean(SD)						
Yes	3.2(3.1)	2.9(2.9)	3.1(3.0)**	4.8(2.7)	4.5(2.7)	4.7(2.7)**
No	3.6(3.1)	3.4(3.1)	3.5(3.1)***	5.5(2.6)	5.3(2.7)	5.5(2.6)***
Personal autonomy *r*	-0.33	-0.33	-0.33***	-0.40	-0.42	-0.41***
Satisfaction with life *r*	-0.28	-0.30	-0.29***	-0.37	-0.39	-0.38***

Abbreviations: SD= Standard Deviation, GDP= Gross Domestic Product, *r*= linear correlation coefficient. [a] For categorical variables, differences in mental health scores between new and old generations for each category were compared by *t* test. For continous variables, linear correlation coefficients between mental health scores and study variables were calculated. Coefficients with asterisks indicated the relationships between mental health scores and study variables were significant in the total sample. *: $P<0.05$; **: $P<0.01$; ***: $P<0.001$.
